# Beyond the blues: marital dissatisfaction as a stronger predictor of postpartum depression than maternity blues among postpartum women: a prospective study in a Turkish cohort

**DOI:** 10.3389/fmed.2026.1769387

**Published:** 2026-01-28

**Authors:** Yusuf Ziya Kızıldemir, Ömer Tammo, Sezin Eda Karslı, Işıl Işık Okuyan, Bekir Kahveci, Sibel Sak, Muhammed Erdal Sak, Helin Kalir, Mehmet İncebıyık, Merve Civelek, Cagri Kutlugun Emral

**Affiliations:** 1 Department of Obstetrics and Gynecology, Harran University, Şanlıurfa, Türkiye; 2 Department of Obstetrics and Gynecology, Şanlıurfa Training and Research Hospital, Şanlıurfa, Türkiye; 3 Department of Nursing, Institute of Health Sciences, Harran University, Şanlıurfa, Türkiye

**Keywords:** maternity blues, perinatal mental health, postpartum depression, postpartum women, propensity score matching

## Abstract

**Purpose:**

This study aimed to determine whether maternity blues (MBs) act as an independent predictor of postpartum depression (PPD), after controlling for key psychosocial factors in a Turkish cohort.

**Methods:**

In this prospective cohort study, 324 women were followed for 6 weeks postpartum. MB status was assessed in the first week, and PPD risk was evaluated at 4–6 weeks via the Edinburgh Postnatal Depression Scale (EPDS; cutoff ≥12). A propensity score matching (PSM) analysis was conducted to control for confounding variables, followed by multivariate logistic regression on the matched cohort. The study was reported in accordance with the STROBE (Strengthening the Reporting of Observational Studies in Epidemiology) guidelines.

**Results:**

Of the 324 participants, 84 (25.9%) were at high risk for PPD. In the matched cohort (*n* = 168), multivariate analysis confirmed MB as a significant independent predictor (adjusted odds ratio [aOR] = 3.52, 95% CI: 1.98–6.24, *p* < 0.001). Critically, marital dissatisfaction emerged as a stronger independent predictor (aOR = 5.98, 95% CI: 2.95–12.14, *p* < 0.001) than maternity blues.

**Conclusion:**

While MB is a significant predictor, its standalone accuracy is modest (AUC = 0.62). Marital dissatisfaction is a more powerful determinant in this cohort. Screening should integrate psychosocial risk factors alongside MB symptoms.

## Highlights

- Marital dissatisfaction was identified as a stronger independent predictor of postpartum depression risk than maternity blues in this Turkish cohort.
- Maternity blues remains a significant independent predictor, though its standalone predictive accuracy is modest (AUC=0.62).
- Propensity score matching confirmed this association after key demographic and clinical confounders were balanced.
- The findings support a two-stage screening protocol for postpartum depression prevention in clinical practice.

## Introduction

The postpartum period is a critical phase of profound physical and emotional changes, representing a time of heightened vulnerability of women to psychiatric disorders (1). Among the mood disturbances that can occur during this period, maternity blues (MBs) and postpartum depression (PPD) are of significant clinical importance. PPD is a global public health concern (2), and its prevalence in Turkey is notably high. Meta-analyses suggest a rate of approximately 24%, whereas individual studies report rates ranging from 21 to 36.7%, underscoring the topic’s significance (3). In Turkey, traditional gender roles and a lack of mental health awareness may exacerbate PPD risk (4). This high prevalence in Turkey may be linked to specific cultural factors, such as societal pressures on new mothers, and the complex role of the extended family, which can be both a source of support and stress (5). Furthermore, barriers to accessing mental health services in some regions can contribute to the underdiagnosis and undertreatment of perinatal mood disorders (6).

Maternity blues are self-limited conditions that occur in the first postpartum week, with symptoms such as crying spells, mood swings, and irritability (1). It usually resolves spontaneously within 10–14 days and is often viewed as part of a normal adaptation process (7). The underlying pathophysiology of MB is believed to involve increased sensitivity to dramatic hormonal fluctuations during the early postpartum period. The sharp decline in estrogen and progesterone may impact neurotransmitter systems, particularly serotonin, which is crucial for mood regulation (8). This neuroendocrine vulnerability could explain why some women experience transient mood disturbances during this period of significant biological adjustment. In contrast, PPD is a more severe and persistent mood disorder. It usually begins within the first 4–6 weeks postpartum and presents with symptoms meeting the criteria for a major depressive episode (e.g., persistent sadness, anhedonia, or functional impairment), requiring clinical intervention (9, 10).

Prospective cohort studies and meta-analyses have consistently demonstrated that MB is a significant risk factor for PPD (8). Indeed, a comprehensive meta-analysis by Beck revealed a moderate correlation (r ≈ 0.35–0.37) between the two conditions (11). This statistical link strengthens the hypothesis that MB may be more than a benign state, potentially serving as a precursor to PPD. However, PPD has a multifactorial etiology, with numerous psychosocial factors, such as marital dissatisfaction, lack of social support, and economic difficulties, that also influence risk. Specifically, factors related to the marital relationship, such as low satisfaction and conflict and the absence of a strong social support network, are consistently cited as some of the most powerful determinants of PPD, particularly within the Turkish cultural context (6). The critical question that remains to be answered is how strongly an MB plays a role as an independent predictor within this complex network of risk factors. Furthermore, current evidence from Turkey remains limited regarding the predictive strength of MB after controlling for social factors such as marital satisfaction and financial stressors. In the Turkish sociocultural structure, marital satisfaction is not merely a personal preference but a fundamental pillar of social support. Marital dissatisfaction often acts as a significant confounding factor in perinatal mental health studies because it directly influences a woman’s resilience to hormonal shifts and the stress of new motherhood. We prioritized this factor as a potential confounder because evidence suggests that in collective cultures, the quality of the spousal relationship can either buffer or exacerbate the psychological impact of early postpartum emotional disturbances.

Therefore, the purpose of this study was twofold: first, to confirm if maternity blues (MBs) remain an independent predictor of PPD risk within a Turkish cohort using a robust matching methodology; and second, to directly compare the predictive strength of MB against key psychosocial factors (e.g., marital dissatisfaction, financial stress) that are culturally salient in Turkey. We hypothesized that while MB would be a significant predictor, psychosocial stressors, particularly marital dissatisfaction, would emerge as equally or more powerful determinants, highlighting the need for a culturally-contextualized, multifactorial screening approach.

## Materials and methods

### Study design and participants

This study employed a prospective cohort design. The study design and reporting followed the STROBE checklist for prospective cohort studies. Women who gave birth at the Department of Obstetrics and Gynecology of the Harran University Faculty of Medicine between January 2023 and December 2024 were prospectively enrolled. The sample size was calculated using G*Power 3.1. Based on previous literature suggesting a medium-to-large effect size for the link between maternity blues and PPD [e.g., Beck’s meta-analysis where (OR ≈ 3.5), we calculated that 320 participants were required to achieve 80% power at *α* = 0.05].

Data from a total of 324 participants who completed the follow-up were included. Inclusion criteria were: (1) aged 18–45 years, (2) giving birth to a singleton live infant, and (3) ability to read and speak Turkish. Exclusion criteria were: (1) history of major depressive disorder or psychosis prior to pregnancy, (2) severe obstetric complications (e.g., massive hemorrhage), or (3) neonates with major congenital anomalies requiring intensive care, as these factors independently increase depression risk and could mask the specific predictive role of maternity blues.

### Cohort enrollment and follow-up

Demographic, clinical and psychosocial data were collected from patient files and self-administered questionnaires at baseline. Exposure to maternity blues was assessed via the Kennerley-Gath blues questionnaire during the first postpartum week. All participants were followed up, and the primary outcome, the risk of postpartum depression (PPD), was determined between the 4th and 6th weeks postpartum via the Edinburgh Postnatal Depression Scale (EPDS). For the purpose of analysis, the participants were categorized according to their EPDS results: women with an EPDS score ≥ 12 constituted the high-risk PPD group, whereas those with a score <12 formed the low-risk PPD group. This grouping was intended to operationally define the high- and low-risk groups in terms of the symptomatology of PPD rather than to establish a clinical diagnosis.

### Measures

- Kennerley-Gath Blues Questionnaire: This is a 28-item self-report scale designed to assess the emotional state of postpartum women. It is answered in a “yes” or “no” format. In this study, it was used to determine the presence of maternity blues. This scale was chosen because it was specifically developed to detect maternity blues in the early postpartum days and its validity and reliability for the Turkish population have been confirmed (12). The scale consists of 28 items, with higher scores reflecting a higher intensity of maternity blues symptoms. In the current study, the internal consistency (Cronbach’s alpha) was found to be 0.88.
- Edinburgh Postnatal Depression Scale (EPDS): This is an internationally validated 10-item scale used to screen for symptoms of postpartum depression. A cutoff score of ≥12 was used to identify a high risk of PPD, as validated in Turkish populations, with 85% sensitivity and 80% specificity (13). It is important to emphasize that this cutoff score identifies women at “high risk” for PPD and serves as a screening outcome, not a formal clinical diagnosis, in line with the scale’s intended use. Each item is scored from 0 to 3, with a total possible score of 30. A higher total score indicates more severe depressive symptoms. The Cronbach’s alpha for the EPDS in our cohort was 0.84.
- Psychosocial Predictors: Key psychosocial variables included in the multivariate analysis (marital dissatisfaction, recent conflicts with partner, financial problems) were assessed via direct, single-item questions during the baseline questionnaire (e.g., “Do you feel dissatisfied with your marital relationship?” answered yes/no).

### Statistical analysis

We conducted all analyses via SPSS Statistics (Version 22.0, IBM Corp.), with statistical significance set at *p* < 0.05 (two-tailed). Baseline characteristics were summarized via descriptive statistics—means±SDs for continuous variables and frequencies/percentages for categorical variables. We compared the high-risk (EPDS≥12) and low-risk (EPDS<12) groups via independent *t* tests for continuous variables and chi-square tests for categorical variables. To address confounding factors, we performed propensity score matching (PSM) through multivariable logistic regression, generating propensity scores that included age, gravida, parity, education, socioeconomic status, and psychiatric history. Partner support was excluded due to collinearity with marital dissatisfaction (VIF > 5), although marital dissatisfaction itself was retained in the final models. The primary psychosocial variables (marital dissatisfaction, partner conflicts, financial problems) were intentionally not included in the PSM algorithm. The PSM was designed to balance baseline demographic and clinical confounders (e.g., age, parity, psychiatric history). The key psychosocial variables were reserved for inclusion in the final multivariate logistic regression model. This approach allowed us to directly test their independent predictive strength relative to maternity blues in a cohort already balanced for baseline characteristics. Using 1:1 nearest-neighbor matching with a 0.2 SD caliper, we matched 84 high-risk participants with 84 low-risk participants, confirming balance through covariate reassessment. In the matched cohort, we performed multivariate logistic regression with EPDS≥12 as the outcome variable. The model included maternity blues as the primary predictor along with significant univariate predictors (marital dissatisfaction, partner conflicts, financial problems), reporting adjusted odds ratios (aORs) with 95% CIs. We assessed multicollinearity (VIF < 2 acceptable) and model fit via Hosmer–Lemeshow tests. Finally, we evaluated the predictive performance of maternity blues through receiver operating characteristic (ROC) curve analysis and calculated the area under the curve (AUC).

### Ethical considerations

Approval for the study was obtained from the Harran University Clinical Research Ethics Committee (Approval No: HRÜ/2023.01.22, Date: 14.01.2023), and informed consent was obtained from all participants for the use of their data in scientific research. In accordance with ethical best practices, participants who scored ≥12 on the EPDS were informed of their results and formally referred to the hospital’s psychiatric department for further evaluation and support.

## Results

### Participant demographics and prematching status

Among the 324 participants included in the cohort, 84 (25.9%) were assigned to the high-risk PPD group and 240 (74.1%) were assigned to the low-risk group on the basis of their EPDS scores. Before propensity score matching (PSM), there were statistically significant differences between the groups in terms of baseline demographic and clinical variables (Table 1).

**Table 1:** Comparison of baseline demographic and clinical variables between high- and low-risk PPD groups before and after propensity score matching.

Variable	Pre-matching high-risk PPD (*n* = 84)	Pre-matching low-risk PPD (*n* = 240)	*p*-value (pre)	Pre-matching SMD	Post-matching high-risk PPD (*n* = 84)	Post-matching low-risk PPD (*n* = 84)	*p*-value (post)	Post-matching SMD
Age (mean ± SD)	29.3 ± 4.5	26.7 ± 5.2	0.02	0.52	28.9 ± 4.3	29.1 ± 4.4	0.85	**0.05**
Gravida (mean)	2.3 ± 1.1	3.0 ± 1.4	0.03	0.60	2.5 ± 1.0	2.6 ± 1.1	0.78	**0.08**
Parity (mean)	2.0 ± 0.8	2.5 ± 1.2	0.04	0.45	2.1 ± 0.7	2.2 ± 0.8	0.90	**0.06**
Education (% high school+)	65%	45%	0.01	0.41	62%	60%	0.95	**0.04**
Socioeconomic status (low %)	55%	70%	0.03	0.31	58%	57%	0.88	**0.02**
Previous psychiatric diagnosis (%)	20%	10%	0.02	0.28	18%	19%	0.92	**0.03**
Low partner support (%)	40%	50%	0.05	0.20	42%	41%	0.98	**0.01**

SMD, standardized mean difference. “Bold” indicates SMD <0.1, confirming excellent balance after matching. SD, standard deviation. Continuous variables are presented as Mean ± SD; categorical variables are presented as percentages (%).

### Effect of propensity score matching

As a result of PSM, a group of 84 high-risk participants was matched with 84 low-risk participants. All postmatching SMDs were <0.1 (Table 1), indicating successful balance across covariates.

### Relationship between maternity blues and postpartum depression

In the analysis conducted on the matched (balanced) cohort, 61.9% (*n* = 52) of the women in the high-risk group had previously experienced maternity blues, whereas 38.1% (*n* = 32) of the women in the low-risk group did. The multivariate logistic regression analysis confirmed that maternity blues significantly increased the odds of being in the high-risk PPD group (adjusted odds ratio [aOR] = 3.52, 95% CI: 1.98–6.24, *p* < 0.001). Other significant independent predictors identified in the model were marital dissatisfaction (aOR = 5.98), recent conflicts with a partner (aOR = 2.45), and financial problems (aOR = 3.42) (Table 2). The adjusted odds ratio (aOR) for marital dissatisfaction was included to highlight its primary predictive role.

**Table 2:** Predictive factors in the multivariate analysis of postpartum depression.

Factors	Adjusted odds ratio (aOR)	95% confidence interval (CI)	*p*-value
Marital dissatisfaction	5.98	2.95–12.14	<0.001
Recent conflicts with partner	2.45	1.12–5.36	0.03
Recent financial problems	3.42	1.39–8.43	0.01
Maternity Blues	3.52	1.98–6.24	<0.001

Multicollinearity was acceptable (VIF range: 1.2–1.8). The severity subgroup analysis (mild vs. severe MB) is exploratory as it was not pre-specified.

To explore a potential dose-dependent relationship, a *post hoc* subgroup analysis based on MB severity was conducted. Within the matched cohort, participants with MBs were categorized according to their Kennerley-Gath scores into “mild MB” (score 8–15) and “severe MB” (score >15) groups. The multivariate analysis revealed that while mild MB was associated with a significant risk of PPD (aOR = 2.48, 95% CI: 1.10–5.59, *p* = 0.02), severe MB was associated with a much stronger risk (aOR = 5.91, 95% CI: 2.85–12.25, *p* < 0.001), suggesting that the severity of early symptoms is a critical determinant of later PPD risk. This finding should be interpreted with caution. As this was a non-prespecified, exploratory *post hoc* analysis, the observed dose–response relationship is susceptible to inflated error rates from multiple testing and requires rigorous validation in future, larger studies designed specifically to test this hypothesis.

**Figure 1:**
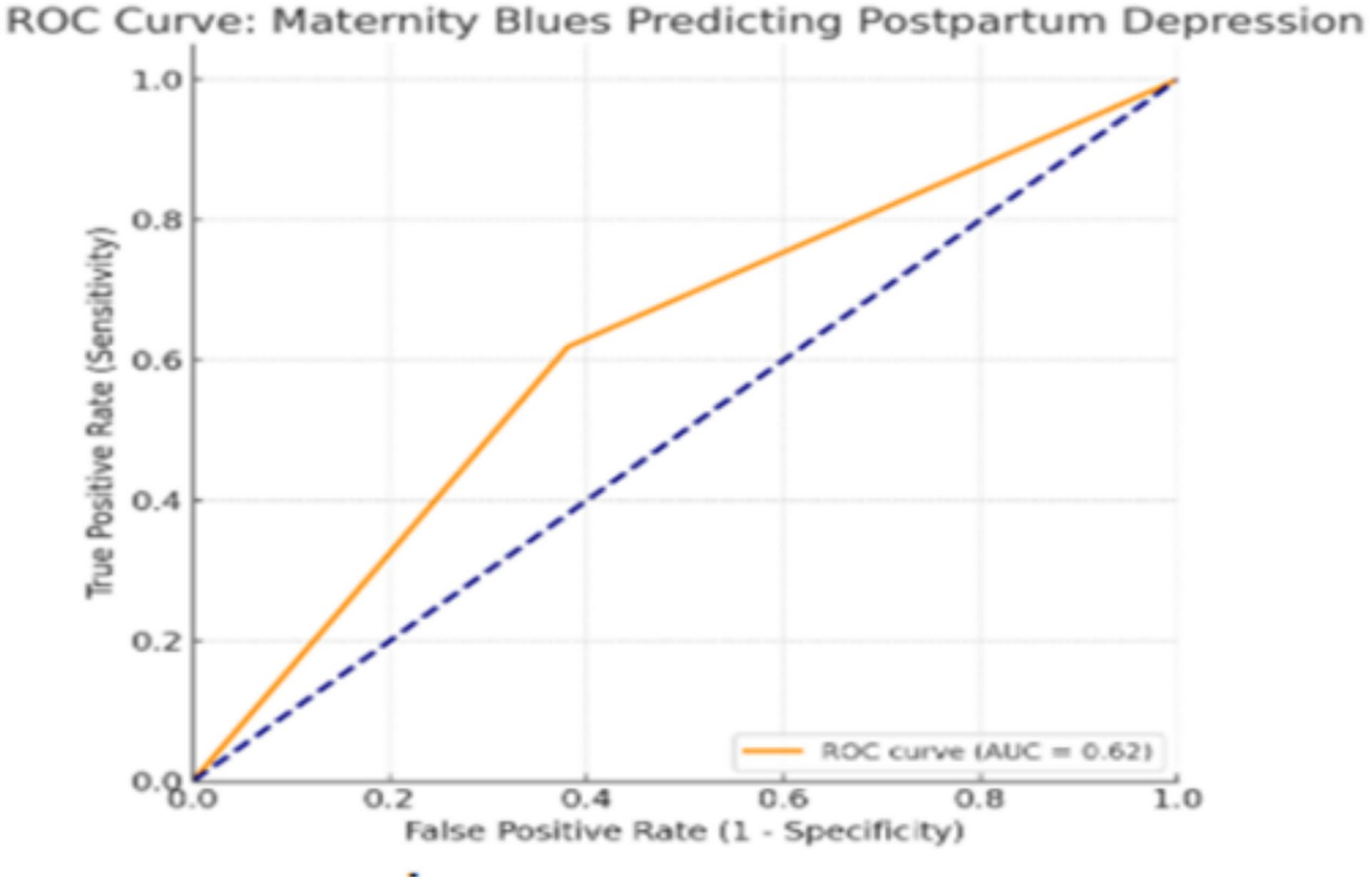
Receiver operating characteristic (ROC) curve for maternity blues predicting high PPD risk in the matched cohort (*n* = 168), AUC = 0.62 (95% CI, 0.55–0.69).

To further evaluate the predictive performance of maternity blues (MBs) for postpartum depression (PPD), a receiver operating characteristic (ROC) curve analysis was performed using the MB status as the binary classifier. The resulting area under the curve (AUC) was 0.62. This value indicates modest discriminative ability and strongly confirms that while MB is a statistically significant predictor, it lacks the accuracy to be used as a standalone screening or diagnostic tool. Instead, it must be integrated into a broader, multifactorial risk assessment framework (Figure 1).

## Discussion

The primary finding of this study is that maternity blues is a statistically significant independent predictor of the likelihood of postpartum depression (PPD) in subsequent weeks, even after controlling for other significant psychosocial and demographic factors, although its discriminative accuracy as a standalone tool was modest (AUC = 0.62). This modest AUC reinforces that while MB is a statistically significant predictor, it lacks the accuracy to be used as a standalone screening tool, aligning with critiques that MB alone is insufficient for robust PPD prediction (14).

The most critical finding of this study, highlighting the specific cultural context, is that marital dissatisfaction (aOR = 5.98) emerged as a substantially stronger predictor of PPD risk than maternity blues (aOR = 3.52). This aligns perfectly with numerous studies conducted in Turkey that identify a lack of social support and spousal issues as among the most powerful determinants of PPD (6). This suggests that in the Turkish context, the quality of the marital relationship and family dynamics may be more powerful determinants of postpartum mental health than the transient biological/emotional changes of MB.

This finding is consistent with the broad international literature identifying maternity blues as a significant risk factor for PPD (15). For example, a prospective study by Watanabe et al. reported that women with severe maternity blues had up to a 9.5-fold increased risk of PPD. In the large French cohort study by Guedeney et al., the risk was found to be 2.1-fold greater (8). The 3.5-fold risk increase found in our study falls within the range of these international findings, strengthening the hypothesis that maternity blues are a universal and quantitatively important predictor for PPD, including in the Turkish population. This finding is consistent with the moderate correlation reported in Beck’s meta-analysis (11) and supports the “dose-dependent” relationship hypothesis, which suggests that not only the presence but also the severity of MB is significant. The fact that not all women with MB develop PPD highlights the complexity of the relationship and the role of other risk factors.

Our findings are compatible with the “continuum” hypothesis, which posits a link between maternity blues and PPD. According to this view, atypical or severe maternity blues may be an early manifestation of an underlying biological (e.g., sensitivity to hormonal changes, HPA axis dysregulation) and psychological vulnerability rather than a simple adaptation process (16). Recent research also points to alterations in inflammatory pathways and oxytocin signaling as potential shared mechanisms (17, 18). However, our findings suggest this continuum is strongly moderated by psychosocial factors. Therefore, it is plausible that PPD is a more severe condition that emerges from the continuation and exacerbation of these early symptoms. This underscores the importance for clinicians to closely monitor patients with severe maternity blues.

This study’s prospective cohort design provides methodological strength by establishing clear temporal relationships while minimizing recall bias, with propensity score matching (PSM) further enhancing confidence through effective balancing of known confounders. However, important limitations must be acknowledged, beginning with the inherent constraints of the EPDS screening tool: our ≥12 cutoff demonstrated only 30.3% positive predictive value in Turkish populations, indicating that most “high-risk” classifications may represent false positives requiring clinical confirmation (13). Beyond this, several other limitations must be considered. First, despite our PSM, we did not control for several key clinical confounders, including a prior history of PPD in previous pregnancies, obstetric or neonatal complications (e.g., emergency C-section, NICU admission), or breastfeeding status, all of which are established PPD risk factors (19–21). Second, a significant methodological limitation is the exclusion of the primary psychosocial predictors (e.g., marital dissatisfaction, financial problems) from the PSM. As justified in our methods, this was done to test their relative strength in the final regression, but it means these powerful confounders were not balanced between the matched groups, and the resulting aORs should be interpreted with caution. Third, as noted in our methods, these key psychosocial predictors were measured using single-item questions rather than validated, multi-item scales (e.g., a formal marital satisfaction inventory), which limits the construct validity and reliability of these measures. Fourth, while we emphasize the Turkish cultural context, our study did not systematically measure specific cultural variables such as family structure, perceived in-law support, or religiosity, which might moderate the MB–PPD pathway. This remains a critical gap for future research. Finally, as a single-center university hospital study, generalizability to primary care or diverse Turkish regions requires caution. Ultimately, the modest standalone predictive value of maternity blues (AUC = 0.62) reinforces the necessity of comprehensive, multifactorial risk assessment in clinical practice.

The findings have direct clinical implications, but they do not support using the 28-item Kennerley-Gath scale as a routine standalone screen, given its modest accuracy (AUC = 0.62) and length. Instead, our results strongly advocate for a two-stage or multifactorial screening protocol. A brief screen for MB symptoms (e.g., even 2–3 key questions) in the first week should not be an end-point, but rather a trigger. A positive MB screen should mandate a second-stage screening focused on the stronger predictors identified: marital dissatisfaction, partner conflict, and financial stress. Interventions should therefore adopt couple/family-focused approaches rather than individual-only models, which is particularly relevant in the Turkish context (22).

## Conclusion

In conclusion, this prospective matched cohort study confirmed that maternity blues are a significant independent predictor of PPD risk in Turkish women. However, its predictive strength is modest (AUC = 0.62), and it was found to be a weaker predictor than marital dissatisfaction. These findings caution against using MB screening in isolation. Instead, they provide strong support for a two-stage screening protocol: (1) early identification of MB symptoms in the first week, which (2) triggers a targeted assessment of key psychosocial factors, particularly the quality of the marital relationship and social support. Future research should focus on validating the clinical utility and cost-effectiveness of this multifactorial approach in the Turkish primary care setting.

## Data Availability

The raw data supporting the conclusions of this article will be made available by the authors, without undue reservation.
